# A myeloperoxidase precursor, pro-myeloperoxidase, is present in human plasma and elevated in cardiovascular disease patients

**DOI:** 10.1371/journal.pone.0192952

**Published:** 2018-03-28

**Authors:** Irada S. Khalilova, Nina Dickerhof, Tessa J. Mocatta, Catriona J. Bhagra, Dougal R. McClean, Christian Obinger, Anthony J. Kettle

**Affiliations:** 1 Centre for Free Radical Research, Department of Pathology, University of Otago Christchurch, Christchurch, New Zealand; 2 Cardiology Department, Christchurch Hospital, Christchurch, New Zealand; 3 Department of Chemistry, Division of Biochemistry, BOKU–University of Natural Resources and Life Sciences, Vienna, Austria; Boston University, UNITED STATES

## Abstract

Myeloperoxidase (MPO)-derived oxidants have emerged as a key contributor to tissue damage in inflammatory conditions such as cardiovascular disease. Pro-myeloperoxidase (pro-MPO), an enzymatically active precursor of myeloperoxidase (MPO), is known to be secreted from cultured bone marrow and promyelocytic leukemia cells, but evidence for the presence of pro-MPO in circulation is lacking. In the present study, we used a LC-MS/MS in addition to immunoblot analyses to show that pro-MPO is present in human blood plasma. Furthermore, we found that pro-MPO was more frequently detected in plasma from patients with myocardial infarction compared to plasma from control donors. Our study suggests that in addition to mature MPO, circulating pro-MPO may cause oxidative modifications of proteins thereby contributing to cardiovascular disease.

## Introduction

The heme peroxidase myeloperoxidase (MPO), a neutrophil-resident bactericidal enzyme, uses hydrogen peroxide to oxidize chloride and thiocyanate to hypochlorous and hypothiocyanous acids, respectively [1, 2]. Bromide is a minor substrate and is oxidized to hypobromous acid [3]. While MPO-derived oxidants play a key role in the innate immune-response by facilitating microbial killing, they have also emerged as a contributor to progressive tissue damage during chronic inflammation, i.e. in cardiovascular disease [4]. Monomeric pro-myeloperoxidase (pro-MPO) is a catalytically active precursor of MPO [5, 6], but whether it is present in human plasma and whether it contributes to cardiovascular disease is unknown.

The biosynthesis MPO involves a complex series of proteolytic processing and trafficking events. Initially, a single primary 80 kDa translation product is expressed in promyelocytes in human bone marrow [7]. Subsequent cleavage of the signal peptide and incorporation of heme and high mannose oligosaccharide then yields the enzymatically active 90 kDa monomeric pro-MPO [8, 9]. After cleavage of the pro-peptide, proteolytic processing produces a 14.5 kDa light and a 59 kDa heavy chain, both of which are covalently bound to the heme. Furthermore, disulfide bond formation between two heavy chains produces mature MPO—a symmetric 147 kDa homodimer [6], which is targeted to azurophilic granules. Mature MPO is released from those granules into the phagosome of stimulated neutrophils [10].

MPO-catalyzed reactions are thought to be involved in all stages of cardiovascular disease, from the initial development of endothelial dysfunction through to the advancement of a mature atherosclerotic plaque and finally its rupture (reviewed in [11]). MPO-derived oxidants can promote lipid peroxidation [12, 13] as well as induce oxidative modifications in proteins including halogenation, nitration and oxidative cross-linking [14, 15]. The presence of 3-chlorotyrosine residues for example has been demonstrated in LDL present in atherosclerotic plaques [16].

Pro-MPO has similar spectroscopic and enzymatic properties as mature MPO, but a lower thermal stability [5, 6, 9]. A recently solved crystal structure of pro-MPO suggests a role for the pro-peptide in aiding proper folding and heme insertion of the enzyme in the endoplasmic reticulum (ER) [6]. It also facilitates transport from the ER to the Golgi apparatus for subsequent proteolytic cleavage [6]. Interestingly, not all of the pro-MPO that is synthesised completes the maturation process to the more thermally stable dimeric MPO. Pro-MPO has been shown to escape proteolytic processing and targeting to the azurophilic granules and to be constitutively secreted by cultured normal bone marrow granulocyte precursors [7] and human promyelocytic leukemia cells (HL-60) [7, 17, 18]. In 1986, Olsen *et al*. also demonstrated the presence of a precursor form in plasma using immunoprecipitation and immunoblot analysis [19]. The apparent molecular weight of the immune-reactive precursor was consistent with pro-MPO, but the sequence of the polypeptide was not identified.

We hypothesized that in addition to circulating MPO, catalytically active pro-MPO can contribute to oxidative modifications of proteins leading to cardiovascular disease. The presence of pro-MPO in patients with cardiovascular disease has not been investigated.

The aim of this study was to confirm the presence of pro-MPO in plasma using LC-MS/MS in addition to immunoblot analyses. Furthermore, we investigated whether pro-MPO was elevated in patients with myocardial infarction (MI).

## Experimental

### Materials

Purified human myeloperoxidase was supplied by Planta Natural Products (Vienna, Austria).

Heterologous expression, purification of recombinant monomeric pro-MPO in CHO cells, and characterization have been described [20, 21]. The concentration of MPO and pro-MPO was determined by measuring its absorbance at 430 nm and using ε_430_ = 89000 M^-1^cm^-1^/heme, and its purity index was calculated by A_430_/A_280_ [22]. NHS-activated Sepharose 4 Fast Flow was purchased from GE Healthcare Life Sciences (Uppsala, Sweden). Rabbit anti-MPO polyclonal antibodies were produced in-house. Antibody production in rabbits was approved by the University of Otago Animal Ethics Committee (# C05/05). One New Zealand white rabbit (5 months old, sourced from Rapid Rabbits) was immunized with human MPO (7.5 μM) injected subcutaneously with Freund’s complete adjuvant (St Louis, MO, USA) at three sites (250 μl/site) at the back of the neck down the spine. Two boosters were injected at 30 day intervals with Freund’s incomplete adjuvant. Test bleeds were from the marginal ear artery and the final bleed via cardiac puncture. The rabbit was given acepromazine for the test bleeds, ice packs were applied and the animal monitored afterwards. Cardiac puncture was carried out under ketamine and xylazine after testing for any response, then cervical dislocation was performed to confirm death. Animals were socialised, handled daily and monitored by staff with daily reports.

All other chemicals were purchased from Sigma (St Louis, MO, USA), BDH (Poole, UK) and Biolab Thermo Fisher Chemicals (Adelaide, Australia).

### Halogenation activity of mature MPO and recombinant pro-MPO

The reaction of MPO-generated hypobromous or hypochlorous acid with NADH was monitored by measuring the formation of the bromohydrin or chlorohydrin product at 275 nm [23]. Initial rates of NADH oxidation were determined with increasing concentrations of bromide (0–20 mM) or chloride (0–300 mM), 20 nM MPO and 100 μM NADH in 50 mM phosphate buffer (pH 7.4) at 21°C. NADH solutions were prepared in 50 mM phosphate buffer, pH 7.4 and concentrations confirmed by absorbance readings at 340 nm using ε_340_ = 6220 M^–1^ cm^–1^.The reaction was started by adding 50 μM H_2_O_2_. Extinction coefficient for the bromohydrin and chlorohydrin products of ε_275_ = 92000 M^–1^ cm^–1^ and ε_275_ = 11000 M^–1^ cm^–1^ were used, respectively [24]. A non-linear hyperbola was fitted to the data using SigmaStat 11 software (Jandel Scientific, San Rafael, CA, USA).

### Plasma samples and isolation of human neutrophils

Blood was collected from healthy human volunteers with written informed consent, and with ethical approval from the Southern Health & Disability Ethics Committee, New Zealand, into tubes containing either EDTA or heparin for obtaining plasma or neutrophils, respectively. EDTA-treated blood was centrifuged at 1200 g for 10 min at 4°C and plasma was separated and stored at -80°C until further processing. Human neutrophils were isolated from heparinized blood by dextran sedimentation followed by Ficoll/Hypaque centrifugation and erythrocyte lysis in hypotonic buffer [25]. Plasma samples from patients with myocardial infarction (MI) were obtained as part of studies described elsewhere and were stored at -80°C [26]. All participants gave written, informed consent.

### HL-60 cells

HL-60 cells were cultured and lysed as described previously with the following modifications [27]. Cells were grown to a density of 1x10^6^ cells/m and harvested by centrifuging 50 ml at 1000 g for 5 min. Cells were re-suspended in buffer A (6.7 mM sodium phosphate buffer pH 6.0, 1 mM MgCl_2_, 3 mM NaCl and protease inhibitors) at a ratio of 10 ml buffer A to 1 ml cell pellet. CHAPS (3-[(3-Cholamidopropyl)dimethylammonio]-1-propanesulfonate) was added to a final concentration of 1% and rotated for 2 h at 4°C. The supernatant was obtained by centrifugation at 20000 g for 20 min at 4°C.

### Immunoprecipitation of MPO from plasma and immunoblot analysis

Rabbit polyclonal anti-human MPO antibodies were produced in-house and precipitated from antiserum by adding saturated ammonium sulphate solution (final concentration of 50%) at 4°C, the pellet was re-suspended in water and dialyzed into PBS. Purified human MPO was covalently coupled to NHS-Sepharose 4 Fast Flow beads following manufacturer’s instructions (0.125 mg of MPO/ml medium). Four ml of the above immunoglobulin fraction was loaded onto 2 ml of MPO-Sepharose and incubated by mixing on rotator overnight at 4°C. The medium was centrifuged at 200 g for 2 min, washed with PBS, eluted with 0.9 ml of 0.1 M glycine, pH 3.0 and the pH was neutralized with 0.1 ml 1M Tris-HCl, pH 8.0. Eluted fractions with an absorbance at 280 nm of 0.03 to 0.5 were combined and buffer-exchanged into PBS using AMICON filters with a 10 kDa molecular weight cut off (Millipore, Billerica, MA). Immobilisation of the purified anti-MPO antibodies on NHS-activated Sepharose 4 Fast Flow beads (about 1 mg of anti-MPO to 2 ml of medium) was performed as above, and used for isolation of immune-reactive MPO from plasma. One ml of plasma diluted in PBS (1:2) was added to 400 μl of washed anti-MPO-Sepharose medium and incubated on a rotator overnight at 4°C. The medium was washed with PBS and MPO was eluted three times by adding 575 μl of 0.1 M glycine, pH 3.0 and neutralized with 25 μl 1 M Tris-HCl, pH 8.0. Eluates were combined and proteins were precipitated by adding 20% [v/v] trichloroacetic acid/ 20% [v/v] acetone/ 0.1% [w/v] sodium deoxycholate. Air-dried precipitates were re-suspended in reducing SDS-PAGE loading buffer (4% [w/v] SDS, 10% [v/v] glycerol, 125 mM Tris-HCl buffer pH 6.5, 0.1% [v/v] β-mercaptoethanol), heated at 95°C for 5 min and then separated by 10% SDS-PAGE. Proteins were transferred to PVDF membranes and incubated with rabbit anti-MPO antiserum produced in-house followed by goat anti-rabbit IgG conjugated to horseradish peroxidase for visualization by the ECL detection system.

### Tryptic digestion and LC-MS analysis

MPO standards or air-dried precipitates obtained after affinity-chromatography purification of plasma samples were solubilized in 100 μl of 6 M guanidine hydrochloride (GuHCl) in 25 mM bicarbonate buffer, pH 8.0 and 5 μl of 200 mM diothiothreitol was added and incubated at 57°C for 1 h. Twenty-five μl of 200 mM IAM was added and incubated in the dark for 30 min at 22°C. GuHCl was diluted to 0.6 M with 25 mM bicarbonate buffer and trypsin (sequencing grade, Promega Corporation, Madison, WI) was added at a ratio of approximately 1/20 (trypsin/protein) and incubated at 37°C overnight. Samples were dried and adjusted to a volume of 60 μl with water containing 0.1% formic acid. Fifty μl of sample was loaded onto an Aries Peptide XB C-18 HPLC column (150 x 2.1 mm, 3.6 μm, Phenomenex, Torrance, CA) for HPLC-MS analysis using a Surveyor HPLC system (Thermo Scientific, San Jose, CA). An acetonitrile gradient from 98% solvent A (0.1% formic acid in water) to 100% solvent B (0.1% formic acid in acetonitrile) was run over 25 min at a flow rate of 200 μl/min. After each gradient, solvent B was held at 100% for 5 min followed by column equilibration over 10 min with 98% solvent A. The HPLC was coupled inline to an electrospray ionization source of a Velos Pro mass spectrometer (Thermo Scientific, Waltham, MA). Voltage was 4 kVolts and nitrogen gas flow was 15 AU. The temperature of the heated capillary was 275°C and vaporizer temperature was 400°C. MRM data were acquired in positive mode between 3.5 and 30 min of each chromatographic run for a pro-MPO and MPO-specific peptides as well as a peptide present in both proteins. The pro-MPO-specific peptide SSGcAYQDVGVTcPEQDK (c, carbamidomethyl-cysteine) and MPO-specific peptide VTcPEQDKYR were detected using the MS1/MS2 transition pairs 1000.9 /1033.5 and 648.8 / 935.5, respectively and the peptide present in both proteins RNGFPVALAR using 551.8 / 272.2 (MS1 = m/z for the doubly charged precursor species and MS2 = m/z for the singly charged y ion fragment). Additionally, full CID-MS/MS spectra were acquired from m/z 200 to 2000 for the precursors (MS1). The window for precursor selection was 1 Da on either side of the m/z; the normalized collision energy was 35 and activation time 30 msec. The longer mature MPO-specific tryptic peptide, VTCPEQDKYR was chosen for SRM analysis over the shorter VTCPEQDK peptide as the latter was not detected.

In some cases, immunoprecipitates were separated by 10% SDS/PAGE under non-reducing conditions as described above. After staining with Coomassie Blue, the 90 kDa band expected to contain pro-MPO was excised from the SDS/PAGE gel and subjected to in-gel digestion with trypsin at a ratio of 1/20 w/w at 37°C overnight. Tryptic fragments were eluted from the gel matrix using 80% acetonitrile, 0.1% FA, dried and taken up in 60 μl water containing 0.1% FA and analyzed by LC-MS/MS as described above.

## Results

### Production of hypobromous and hypochlorous acid by pro-MPO compared to mature MPO

**Fig 1:**
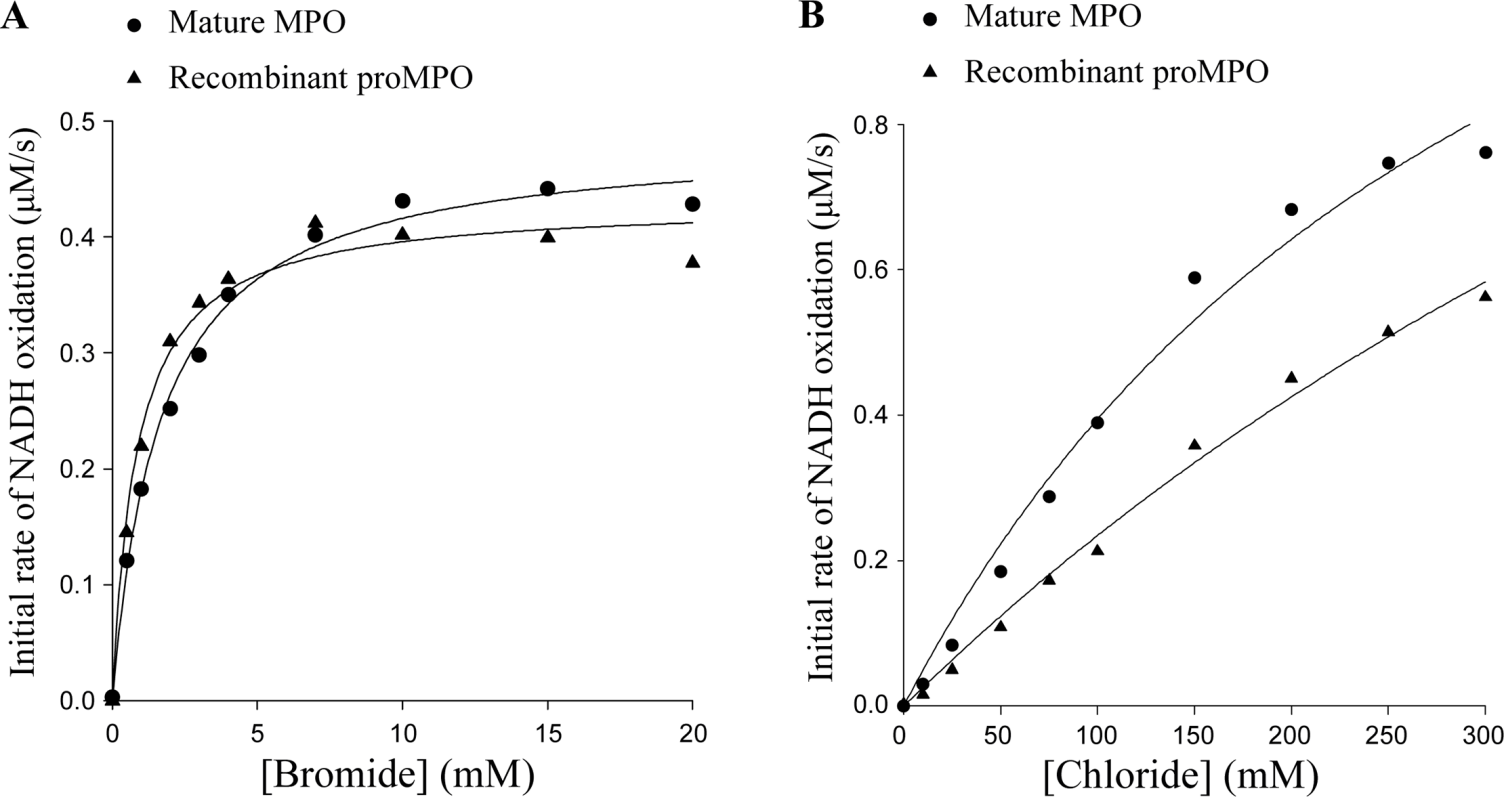
Halogenation activity of mature MPO and recombinant pro-MPO. Initial rates of NADH oxidation were determined with various concentrations of A) bromide or B) chloride, 20 nM mature MPO or recombinant pro-MPO and 100 μM NADH in 50 mM phosphate buffer (pH 7.4) at 21°C. The reaction was started by adding 50 μM H_2_O_2_. The kinetics of the reaction of myeloperoxidase-generated hypobromous and hypochlorous acids with NADH were monitored by measuring the bromohydrin and chlorohydrin product at 275 nm. Data are representative of two or more experiments.

Others have shown that pro-MPO oxidizes chloride and bromide at similar rates to the mature enzyme [5]. We wanted to confirm these results and show that hypohalous acids are produced by pro-MPO. To achieve this, we determined the rate of oxidation of NADH by the MPO/H_2_O_2_/halide system, which is known to produce halohydrins [23]. Recombinant monomeric pro-MPO showed very similar bromination activity as mature MPO as demonstrated by similar rates for the hypobromous acid-dependent oxidation of NADH (Fig 1). The chlorination activity of pro-MPO was about 70% of that of mature MPO.

### Detection of pro-MPO in plasma using affinity purification and immunoblot analysis

**Fig 2:**
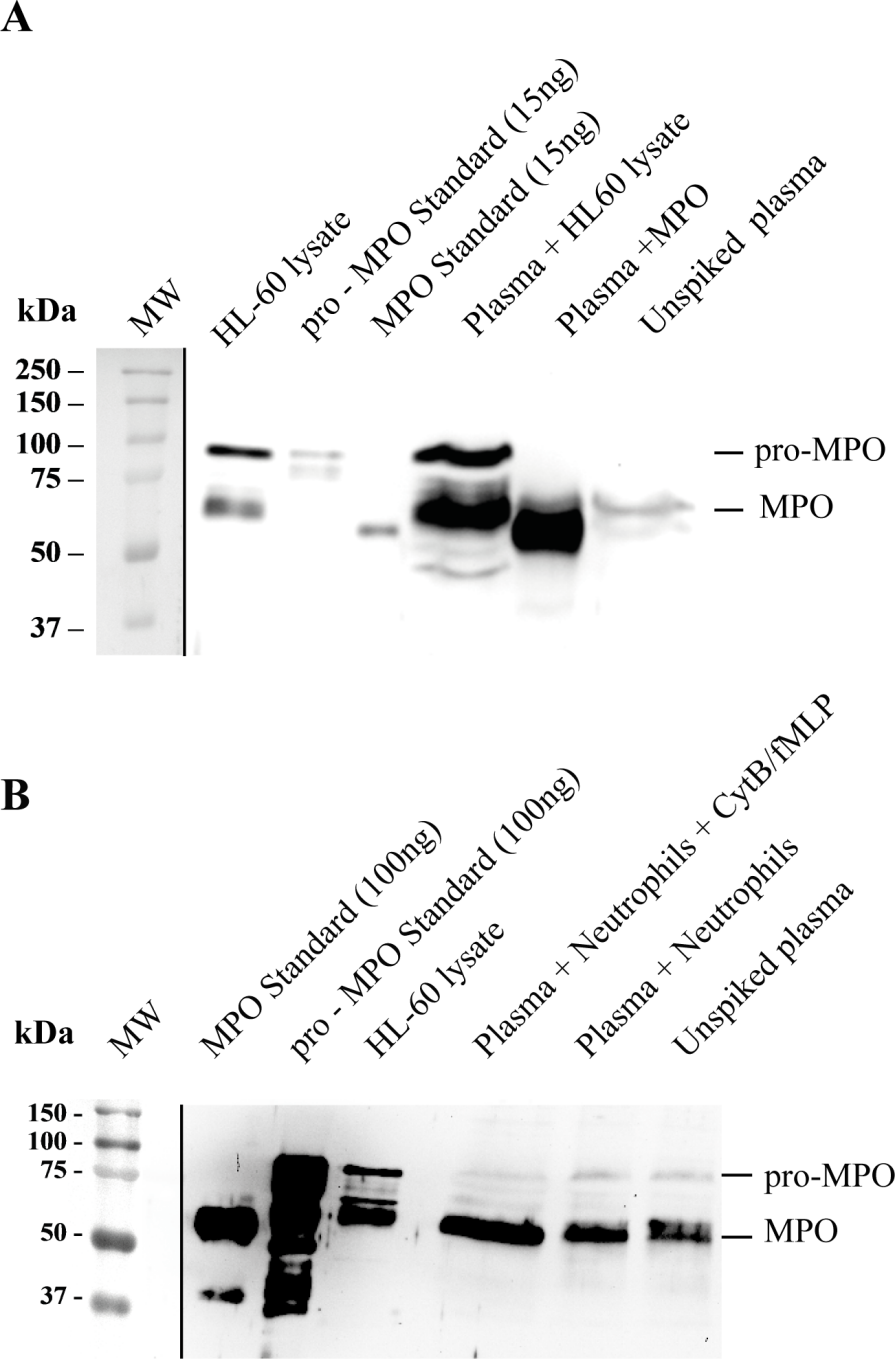
Detection of mature MPO and pro-MPO in plasma using affinity-purification and immunblot analysis. **A)** Plasma was spiked with MPO standard (10 nM) and HL60 cell lysate (containing 10 nM MPO) and subjected to affinity purification. Purified samples along with MPO, pro-MPO standards and HL-60 lysates were separated by 10% SDS/PAGE, transferred to PVDF and probed with MPO-specific antibody. **B)** Neutrophils (5x10^6^ cells/ml) were added back into plasma and then stimulated with CytB and FMLP for 30 min at 37°C. Neutrophils were centrifuged and cell free plasma MPO was subjected to affinity purification and analyzed as described in A. After the ECL fluorescence of blots was developed, a photograph of the blot showing the molecular weight markers was taken and aligned with the fluorescence image as indicated by the black line.

To isolate MPO from human plasma, we purified MPO-specific antibodies from rabbit antiserum produced in-house and immobilized the antibodies on Sepharose. First, we tested whether anti-MPO-Sepharose could isolate pro-MPO and mature MPO from control plasma spiked with purified human mature MPO and HL-60 cell lysate containing both mature and pro-MPO [7] (Fig 2A). Spiked mature and pro-MPO were successfully recovered from plasma using anti-MPO-Sepharose. Interestingly, a protein with the electrophoretic mobility of mature MPO was also isolated from unspiked plasma, but none with the molecular weight of pro-MPO.

Next, we tested whether neutrophils release pro-MPO when added back into control plasma and stimulated with FMLP. While we observed an increase in the intensity of the mature MPO band, pro-MPO was not released into plasma by stimulated neutrophils (Fig 2B).

**Fig 3:**
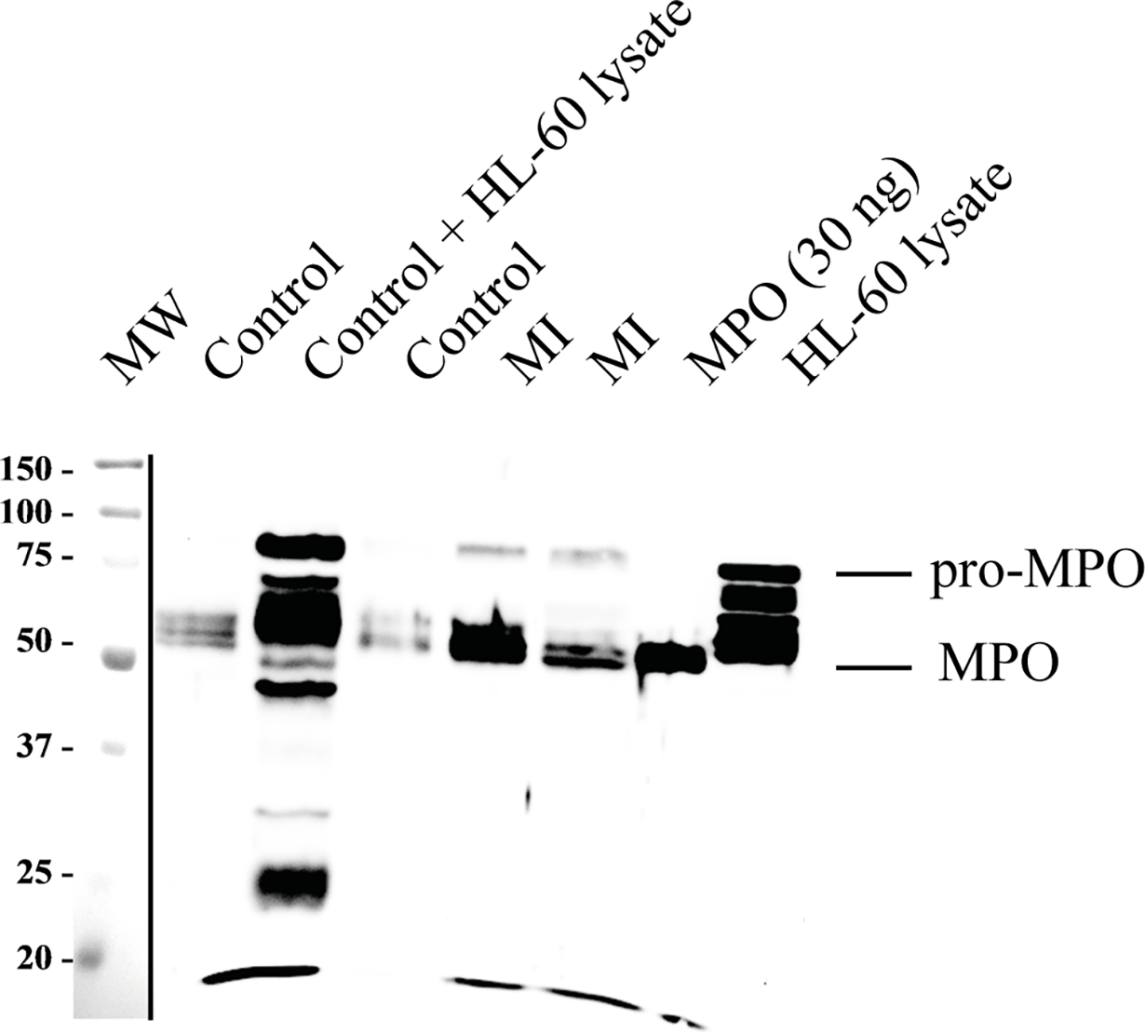
Detection of mature MPO and pro-MPO in plasma from healthy controls and patients with myocardial infarction using affinity-purification and immunblot analysis. Plasma from control subjects and from patients with myocardial infarct (MI) was subjected to affinity purification and analyzed by immunoblot as described Fig 1. This blot is representative of at least 10 plasma samples in each group. After the ECL fluorescence of blots was developed, a photograph of the blot showing the molecular weight markers was taken and aligned with the fluorescence image as indicated by the black line.

We then used anti-MPO-Sepharose to purify immune-reactive MPO from plasma from patients with myocardial infarction and from healthy controls (Fig 3). Bands consistent with the molecular weight of both mature MPO and pro-MPO were detected in plasma from controls and patients. Both bands were more intense in plasma from patients compared to controls (Fig 3). We affinity-purified ten samples in each of the control and myocardial infarction and subsequent immunblot analysis showed both bands to be consistently elevated in the clinical samples. Some healthy volunteers donated control plasma for affinity purification and immunblot analysis on more than one occasion. The intensity of the pro-MPO and mature MPO relative to each other varied suggesting a different physiological source or turn-over for the two proteins.

### Detection of pro-MPO in plasma using affinity purification and LC-MS analysis

**Fig 4:**
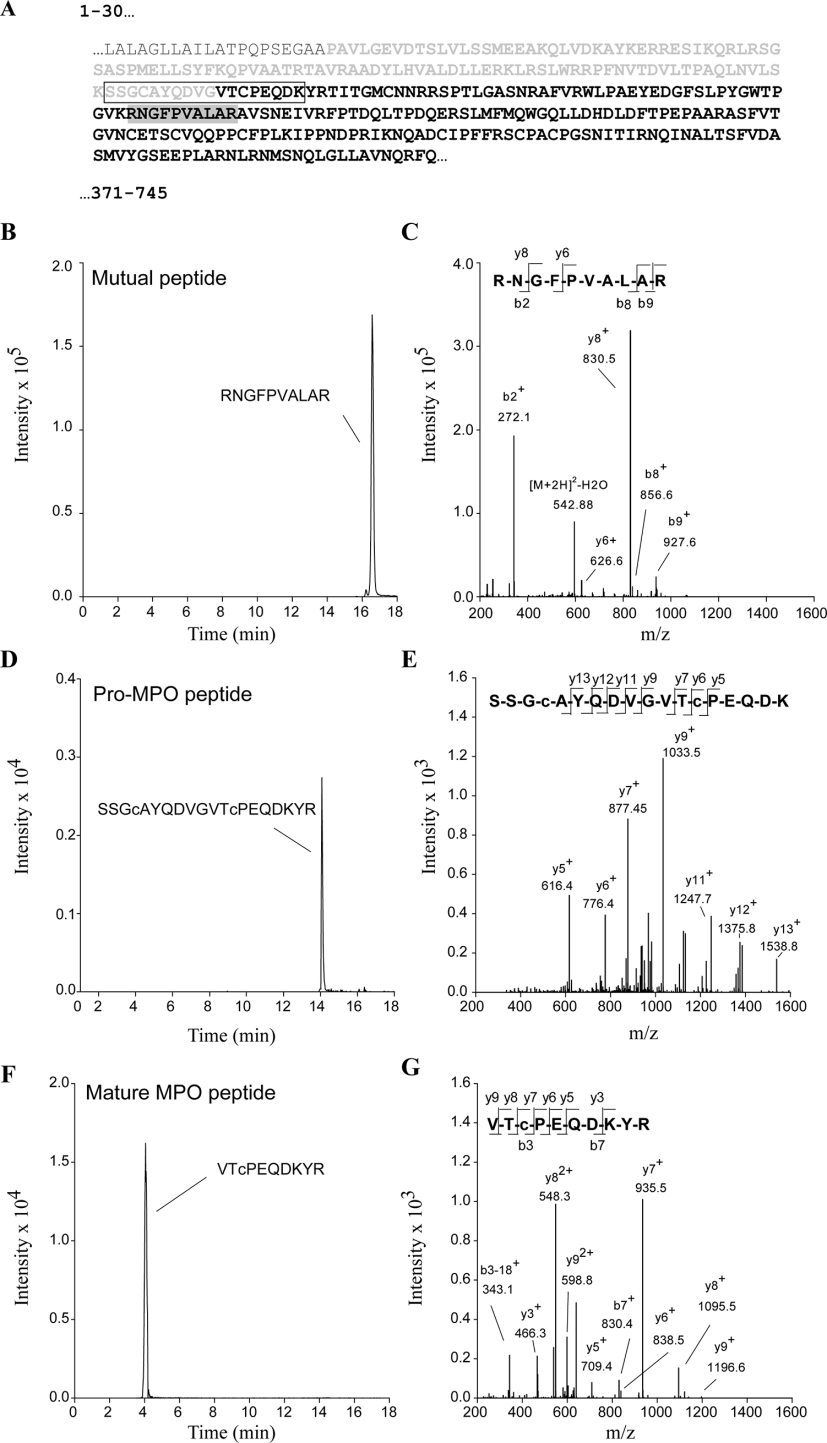
LC-MS/MS analysis of mutual, pro-MPO- and MPO- specific peptides. **A)** Location of tryptic peptides used for LC-MS/MS analysis within the MPO sequence. The *N-*terminal signal peptide is shown in dark grey, the pro-peptide in bold light grey and the sequence of mature MPO in bold black. Highlighted by the black and grey boxes are tryptic peptides specific to pro-MPO or present in both mature MPO and pro-MPO, respectively. Twenty-five μg of recombinant pro-MPO or mature MPO were digested with trypsin and analyzed by LC-MS/MS. **B), D) +F)** Extracted ion chromatograms for SRM transitions specific for the mutual, pro-MPO and mature MPO-specific peptides (MS1/MS2 551.8/272.2, 1000.9/1033.5 and 648.8/935.5, respectively, MS1 = m/z for the doubly charged precursor species and MS2 = m/z for the singly charged y–ion fragment, c–carbamidomethyl-cysteine). **C), E) +G)** CID-MS/MS spectra confirming the sequence of the respective peptide. Representative chromatograms and spectra are shown.

To confirm that the 90 kDa band observed in immunoblots corresponds to pro-MPO and that it is elevated in patient samples, we developed an LC-MS/MS method for the detection of a tryptic peptide specific for pro-MPO, mature MPO as well as a peptide present in both species. The peptide amino acid sequences of the chosen peptides and their location within the MPO sequence is shown in Fig 4A. All three peptides were separated by C-18 chromatography (Fig 4B, 4D and 4F) and collision induced (CID)-MS/MS spectra confirmed the amino acid sequence of each peptide (Fig 4C, 4E and 4G). From these spectra, SRM transitions specific for each peptide were chosen for subsequent SRM analyses of in-gel and in-solution tryptic digests of plasma samples purified by affinity chromatography. The signal intensity for the mutual peptide was 10-fold higher compared to the pro-MPO or mature MPO specific peptides.

Using the SRM-based LC-MS/MS method, we investigated the recovery of the pro-MPO-, mature MPO-specific and the mutual peptide after affinity purification and tryptic digestion of plasma spiked either 90% MPO and 10% pro-MPO or 10% MPO and 90% pro-MPO (Table 1). The LC-MS signal for the pro-MPO- and mature MPO- specific peptides reflected the spiked protein ratios and the signal for the mutual peptide was similar in the two preparations. These results indicate that the MPO-specific antibodies used for affinity purification have a similar affinity to both mature MPO and pro-MPO.

**Table 1:** Recovery of the pro-MPO-, MPO-specific and mutual peptide after affinity purification, tryptic digestion and detection by LC-MS/MS. Plasma was spiked with one μg of total MPO containing either 90% MPO and 10% pro-MPO or vice versa, subjected to affinity purification with MPO-specific antibodies, digested with trypsin and analyzed by SRM-based LC-MS/MS. The area under the curve (AUC) for the pro-MPO-specific peptide, mature MPO-specific peptide and the mutual peptide are shown (see Fig 4). Data are presented as means ± range of two independent experiments.

% Pro-MPO / % mature MPO	AUC of peptide specific for		
	Mature MPO x 10^2^	Pro-MPO x 10^2^	Both x 10^4^
10 / 90	0.73 ± 0.12	0.34 ± 0.05	4.29 ± 0.17
90 / 10	0.19 ± 0.04	3.40 ± 0.26	3.91 ± 0.16

**Fig 5:**
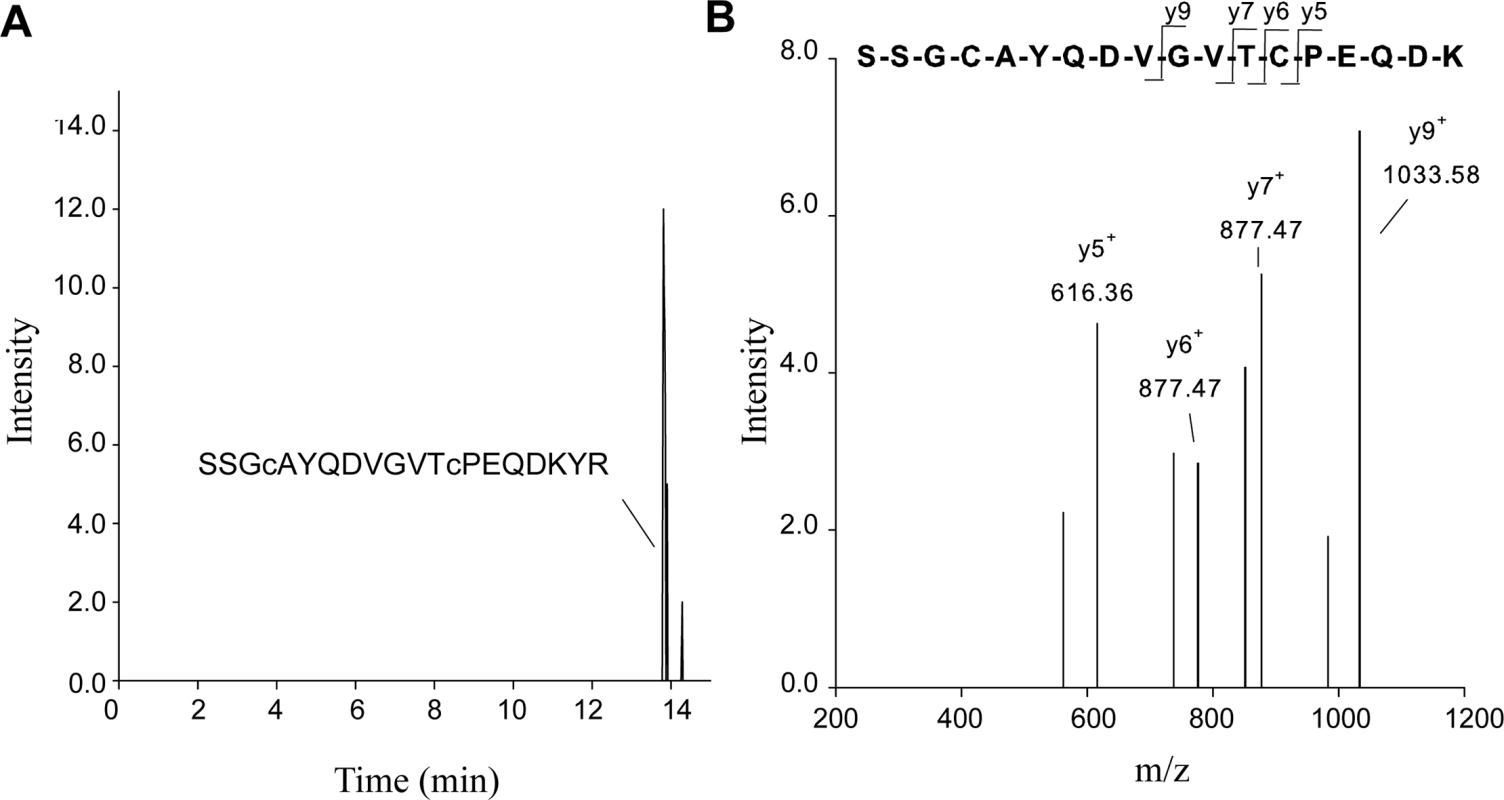
LC-MS/MS detection of pro-MPO in affinity-purified plasma separated by SDS/PAGE. Plasma was purified by affinity chromatography with an MPO-specific antibody and subjected to separation by SDS/PAGE. Bands with the molecular weight of pro-MPO (90 kDa) were subjected to in-gel tryptic digestion and analyzed by LC-MS/MS using SRM for a pro-MPO-specific peptide (1000.9->1033.5). **(A)** Extracted ion chromatogram for the SRM transition for the pro-MPO specific peptide. **(B)** CID-MS/MS spectrum confirming the sequence for the pro-MPO-specific peptide SSGcAYQDVGVTcPEQDK. Representative chromatograms and spectra are shown.

Next, we wanted to confirm by in-gel tryptic digestion that the protein migrating at the molecular weight of pro-MPO (90 kDa) observed in immunoblots of affinity-purified plasma samples contained the pro-MPO-specific peptide. However, protein staining of the corresponding SDS/PAGE gel with coomassie did not show a 90 kDa band indicating that the amount of protein present in the gel was below the limit of detection of our mass spectrometer. Due to limited sample availability, we were unable to scale up the affinity-purification of clinical plasma samples. In contrast, control plasma samples were obtained fresh for the purpose of this study, and we were able to increase the amount of control plasma used for affinity-purification by 25-fold. We obtained a corresponding 90 kDa coomassie-stained band and in-gel tryptic digestion showed it to contain the pro-MPO specific tryptic peptide (Fig 5).

**Fig 6:**
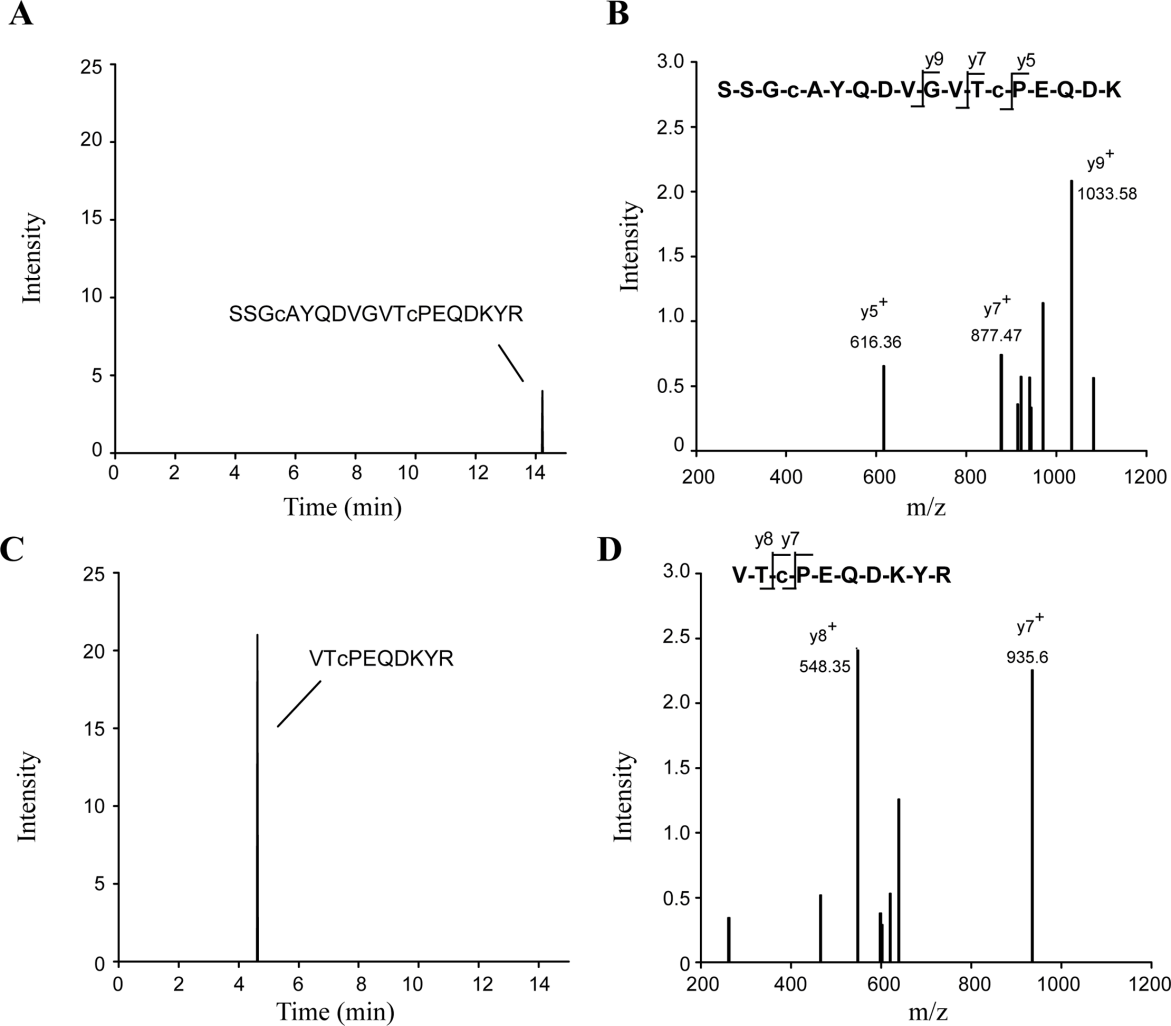
LC-MS/MS detection of pro-MPO and mature MPO in affinity-purified plasma from patients with myocardial infarction (MI). Plasma was purified by affinity chromatography with an MPO-specific antibody, subjected to digestion with trypsin and analyzed by LC-MS/MS. **A)** Representative SRM- based extracted ion chromatogram for the pro-MPO specific peptide (1000.9->1033.5) and **C)** the mature MPO-specific peptide (648.8->935.5) measured in a MI plasma sample. **B)+D)** CID-MS/MS spectra were recorded simultaneously for m/z 1000.9 and 648.8 representing the SSGcAYQDVGVTcPEQDK and VTcPEQDKYR peptides, respectively.

To circumvent likely losses of protein associated with SDS/PAGE and in-gel tryptic digestion, we also performed in-solution digests of immunoprecipitates (Fig 6A–6D). Using this method, the pro-MPO-specific was detected in 25% of plasma samples from patients with myocardial infarction (n = 3/12), but only in 11% of samples from control subjects (n = 1/9). The MPO-specific peptide was detected in 42% of myocardial infarction samples (n = 5/12) and 22% of control samples (n = 2/9) (Table 2).

**Table 2:** Increased detection of pro-MPO in plasma from patients with myocardial infarction (MI) compared to healthy controls. Plasma was purified by affinity chromatography with MPO-specific antibodies, subjected to digestion with trypsin and analyzed for the presence of pro-MPO and mature MPO-specific peptides by SRM-based LC-MS/MS. Samples that showed a peak are denoted by “+”, those that don’t by “-”.

Sample	Peptide specific for	
	Pro-MPO	MPO
Control 1	-	-
Control 2	-	-
Control 3	+	+
Control 4	-	-
Control 5	-	-
Control 6	-	-
Control 7	-	-
Control 8	-	+
Control 9	-	-
MI 1	+	-
MI 2	-	+
MI 3	+	-
MI 4	-	-
MI 5	+	+
MI 6	-	+
MI 7	-	+
MI 8	-	-
MI 9	-	-
MI 10	-	-
MI 11	-	-
MI 12	-	+

## Discussion

In the present study we show by MPO affinity-purification of plasma with subsequent immunoblot and LC-MS/MS analyses that pro-MPO, an enzymatically active precursor of MPO is present in circulation. Our data further shows that circulating pro-MPO, like MPO, is elevated in patients with cardiovascular disease.

Prior to this work, only one study has suggested the presence of pro-MPO in circulation when a 90 kDa band was observed in immunoblot analysis of affinity-purified plasma [19]. Therefore, the physiological and pathological role of the precursor form of MPO, pro-MPO, remains largely unexplored. Mature MPO, in contrast, has been extensively studied with regard to its role in host defence and inflammatory conditions [4]. In this study, we confirm that pro-MPO is present plasma and elevated in cardiovascular disease patients using a combination of immunoblot analysis and LC-MS/MS.

Here we show that pro-MPO has bromination and chlorination activity similar to that of the mature enzyme. A kinetic study previously compared pro-MPO and mature MPO purified from human blood using multi-mixing stopped flow technique, in which the redox intermediates of these two enzymes were found to be indistinguishable [5]. A recent crystal structure confirms that the active site structure remains conserved during the maturation process from pro-MPO to mature MPO [6]. Pro-MPO, like MPO, therefore has the ability to oxidize chloride to hypochlorous acid, an activity that is unique among the mammalian heme peroxidases [28]. We hypothesise that circulating pro-MPO can contribute to oxidative cell damage such as that previously attributed to mature MPO in cardiovascular disease [11].

The source of circulating pro-MPO is elusive and needs further study. Our data suggests that the release of pro-MPO into plasma is independent of neutrophil activation and degranulation as activated neutrophils did not release pro-MPO into plasma. Also, immunoblots of plasma obtained from the same person on different occasions showed that the ratio of pro-MPO to mature MPO is not constant indicating alternative mechanisms for the release of these two enzymes into circulation. MPO is only actively synthesized by promyelocytes and promyelomonocytes during the differentiation process of granulocytes in the bone marrow when azurophilic granules are formed [29, 30]. It has previously been demonstrated in a neutrophil precursor cell line (HL-60) that pro-MPO is secreted into the cell culture medium, but was absent in the granules [7, 17, 18]. Therefore, it seems likely that pro-MPO in plasma originates directly from immature granulocytes in the bone marrow.

Plasma and serum levels of mature MPO correlate well with the total number of neutrophils in the blood indicating that circulating neutrophils are the main source for mature MPO [31, 32]. However, serum MPO has also been suggested to stem directly from the bone marrow during periods of increased myelopoietic activity such as in childhood or during pregnancy [32]. If pro-MPO, unlike MPO, originates exclusively from myelocyte precursors in the bone marrow, then plasma levels of pro-MPO could be explored as marker of increased or ineffective bone marrow activity.

Here, we demonstrate that pro-MPO is elevated in patients with myocardial infarction indicating its prognostic potential that warrants further study. Pro-MPO in plasma from the cardiovascular patients might also stem from tissue macrophages, the prominent cell type in atherosclerotic lesions, where MPO has been shown to be expressed [33]. Under normal conditions macrophages only express little MPO. It is possible that MPO gene transcription has been activated in foam cell macrophages due to the presence of cytokines in the environment of the atherosclerotic plaque. For example, agonists of peroxisome proliferator-activated receptor (PPAR) have shown to regulate MPO gene expression [34–36] and PPAR is abundant in foam cell macrophages [37]. Foam cells undergoing de novo MPO synthesis due to the activation by local cytokines could lack the cellular machinery to process pro-MPO to mature MPO. This would result in constitutive secretion of pro-MPO into circulation and explain why in the present study pro-MPO was more readily detected in patients with cardiovascular conditions.

Finally, we need to acknowledge the limitations of our study. The clinical samples used for the present work were used for earlier studies and had been stored at -80°C for up to five years and freeze-thawed two to three times prior to this work. Therefore, proteins may already have undergone some degradation. Furthermore, the sensitivity of the ion trap mass analyser used for this study is low and the amount of plasma used for the affinity purification could not be increased due to limited sample availability. Collectively, this may explain the very low signal intensity when analysing immunoprecipitates by SRM-based LC-MS/MS and the fact that only a small number of y-ions were detected when full CID-MS/MS spectra were collected. Pro-MPO was detected in a greater percentage of patient samples by immunoblot analysis than by LC-MS/MS indicating the superior sensitivity of the immunochemical method. Consequently, our present data does not allow for any quantitative comparisons between control and disease groups due to the low sensitivity of the LC-MS/MS method, the small number and quality of the patient plasma samples. However, we consider the present study a proof-of concept study that warrants recruiting a greater number of samples using a more sensitive mass spectrometer like a contemporary triple-quad.

## Conclusions

Our study shows that a precursor of MPO, pro-MPO, is present in circulation and elevated in patients with cardiovascular disease. Since enzymatically active, pro-MPO can catalyse the oxidation of proteins and lipoproteins thereby fuelling the progression of an atherosclerotic lesion independent of neutrophil activation and the release of mature MPO from the azurophilic granules. Furthermore, circulating pro-MPO has the potential to be used as a marker of increased bone marrow activity. We hope that these results will stimulate further studies into the role of this enzyme plays in circulation.

## Abbreviations

FMLP: *N*-Formylmethionyl-leucyl-phenylalanine
LC-MS/MS: liquid chromatography with tandem mass spectrometry
MPO: myeloperoxidase
pro-MPO: pro-myeloperoxidase
MI: myocardial infarction
SRM: single reaction monitoring
